# Supplementary material for: Obstacles to university food pantry use and student-suggested solutions: A qualitative study
Source: PLoS One. 2022 May 20;17(5):e0267341. doi: 10.1371/journal.pone.0267341 (PMC9122219; doi:10.1371/journal.pone.0267341)
Supplement: S1 Appendix — (DOCX) [file pone.0267341.s001.docx]

**S1. Interview Guide**

***Obstacles to University Food Pantry Use and Student-suggested Solutions: A Qualitative Study***

# Introduction

Thank you for agreeing to participate in our session today.

My name is X, I'm a graduate student at the University of Florida. The interview we are having today is part of my dissertation project. My colleague X will also be attending this session and will be taking notes of our discussion.

You responded that you have (OR have not) used the on-campus food pantry (identifying users vs. non-users). We have attempted to group these discussions by previous use or not. We’re most interested in learning about why you have (not) accessed the pantry, what barriers stood in your way, and what would help eliminate the barriers you face in accessing the pantry. Information from our discussion will be used to inform on-campus programming to improve student food access. There are no right or wrong answers so I hope that you can express yourself with no hesitation. If there are any questions you prefer not to answer, please feel free not to respond. Also, if you need us to repeat a question, please ask.

I’d like to remind you that we are audio-recording this session so that we do not miss any comments. Please be assured that your responses will be kept anonymous and no one besides the University of Florida research team will be allowed to access the recordings or transcripts. Once the transcription is completed, the recording will be destroyed. This session will take 45-60 minutes.

# Semi-structured interview script

1. Please describe what you know about the campus food pantry. *(Probing: why does it exist on campus? where is it located? what food products are available there? who can use it?)*
2. Tell me about the feelings or thoughts you associate with using the campus food pantry.
3. If you have used the food pantry, tell me about your experience. *(Probing: what was your first impression? what did you think of the food products offered? how was your experience with the staff?)*
4. What foods or food products would make you most likely to use the food pantry?
5. In your opinion, how do others think about you when they know that you use (or plan to use) the food pantry? *(Probing: what would your friends think? would you go alone or with friends? how do they influence your decision?)*
6. In your opinion, what are some things that make it hard use the campus food pantry? *(a- Probing for non-pantry users: what prevented you from using the food pantry so far? b- Probing for users: what prevented you from visiting the pantry more frequently? why do you think other students do not use the pantry?)*
7. What can the university administrators do to make it easier to access the food pantry? *(Probing: can you provide some examples?)*
8. What do you think of how the pantry is advertised? what would you do differently? *(Probing: can you provide some examples of messages that encourage students to use the pantry?)*
9. Is there anything that you’d like to add to what we talked about?

Thank you for your time!
