# Supplementary material for: Obstacles to university food pantry use and student-suggested solutions: A qualitative study
Source: PLoS One. 2022 May 20;17(5):e0267341. doi: 10.1371/journal.pone.0267341 (PMC9122219; doi:10.1371/journal.pone.0267341)
Supplement: S2 Appendix — (DOCX) [file pone.0267341.s002.docx]

**S2.** Consolidated criteria for reporting qualitative studies (COREQ)

| **No.** | **Item** | **Question** | **Description** |
| --- | --- | --- | --- |
| **Domain 1: Research Team and Reflexivity**  *Personal Characteristics* | | | |
| 1. | Interviewer | Which author(s) conducted the interviews? | First author (AE) conducted the interviews. |
| 2. | Credentials | What were the researchers’ credentials at the time of the study? | AE: MS, RD  MV: PhD, MPH KS: PhD, RD KW: PhD AM: PhD, RD |
| 3. | Occupation | What was their occupation at the time of the study? | AE: PhD candidate  MV: Postdoctoral fellow  KS: Associate Professor & Extension Specialist  KW: Professor  AM: Associate Professor |
| 4. | Gender | Was the researcher male or female? | AE: Female  MV: Female  KS: Female  KW: Female  AM: Female |
| 5. | Experience and training | What experience or training did the researcher have? | AE: Training in qualitative research as part of undergraduate and graduate coursework and additional training prior to data collection and analysis:   - Fundamentals of Qualitative Research by Johnny Saldana - Research Methods in the Social Sciences by Bridget Somekh & Cathy Lewin - Using Thematic Analysis in Psychology by Virginia Braun and Victoria Clarke   MV: Formal training in qualitative research and conducts several original qualitative studies.  KS: Leads research studies on food insecurity and food assistance with some experience in qualitative research.  KW: Leads and conducts qualitative research studies and supervised qualitative research by doctoral students.  AE: Conducts original research in qualitative inquiry. |
| *Relationship with Participants* | | | |
| 6. | Relationship established | Was a relationship established prior to study commencement? | There were no pre-existing relationships between participants and the interviewer. |
| 7. | Participant knowledge of the interviewer | What did the participants know about the researcher? | Participants were knowledgeable about the role and status (PhD Candidate at the University of Floroda) of the researcher. |
| 8. | Interviewer characteristics | What characteristics were reported about the interviewer? | The interviewer was a PhD candidate who conducted this study as a part of her dissertation research. |
| **Domain 2: Study Design**  *Theoretical Framework* | | | |
| 9. | Methodological orientation | What methodological orientation was stated to underpin the study? | Qualitative description. |
| *Participant Selection* | | | |
| 10. | Sampling | How were participants selected? | Purposive sampling. |
| 11. | Method of approach | How were participants approached? | Email and in-person. |
| 12. | Sample size | How many participants were in the study? | 41. |
| 13. | Non-participation | How many people refused to participate or dropped out? Reasons? | None. |
| *Setting* | | | |
| 14. | Setting of data collection | Where was the data collected? | Private room at the University of Florida campus. |
| 15. | Presence of non-participants | Was anyone else present besides the participants and researchers? | No. |
| 16. | Description of sample | What are the important characteristics of the sample? | College students enrolled at the University of Florida. Majority are women, undergraduate, and classified as food insecure according to the USDA AFSSM. |
| *Data Collection* | | | |
| 17. | Interview guide | Were questions, prompts, guides provided by the authors? Was it pilot tested? | Yes. The guide was improved/refined throughout the data collection process. |
| 18. | Repeat interviews | Were repeat interviews carried out? | No. |
| 19. | Audio/visual recording | Did the researcher use audio or visual recording to collect the data? | Interviews were audio-recorded. |
| 20. | Field notes | Were field notes made during and/or after the interviews? | Yes. Field notes were made during and immediately following interviews. |
| 21. | Duration | What was the duration of the interviews? | Approximately 45-60 minutes. |
| 22. | Data saturation | Was data saturation discussed? | Yes. |
| 23. | Transcripts returned | Were transcripts returned to participants for comment or correction? | No. |
| **Domain 3: Analysis and Findings**  *Data Analysis* | | | |
| 24. | Number of data coders | How many coders coded the data? | One researcher (AE) coded the data independently. Codes and categories were discussed and refined within the research team. |
| 25. | Description of the coding tree | Did authors provide a description of the coding tree? | Yes. |
| 26. | Derivation of themes | Were themes identified in advance or derived from the data? | Derived from the data. |
| 27. | Software | What software, if applicable, was used to manage the data? | *NVivo* 12. |
| 28. | Participant checking | Did participants provide feedback on the findings? | No. |
| *Reporting* | | | |
| 29. | Quotations presented | Were participant quotations presented to illustrate the themes/findings? Was each quotation identified? | Yes, quotations were included and identified by theme. |
| 30. | Data and findings consistent | Was there consistency between the data presented and the findings? | Yes. |
| 31. | Clarity of major themes | Were major themes clearly presented in the findings? | Yes. |
| 32. | Clarity of minor themes | Is there a description of diverse cases or discussion of minor themes? | Both major and minor themes were discussed. |
