# Supplementary material for: Obstacles to university food pantry use and student-suggested solutions: A qualitative study
Source: PLoS One. 2022 May 20;17(5):e0267341. doi: 10.1371/journal.pone.0267341 (PMC9122219; doi:10.1371/journal.pone.0267341)
Supplement: Attachment — Submitted filename: Response to reviewers 2.23.22.docx [file pone.0267341.s003.docx]

Dear Editor:

We would like to thank our reviewers for a thoughtful critique of our manuscript, “Obstacles to University Food Pantry Use: A Qualitative Study”. Below, we have addressed reviewer’s 1 critique, referencing changes to our manuscript where applicable. We hope these revisions are received favorably by reviewers and the editorial staff.

1. When submitting your revision, we need you to address these additional requirements.

Please ensure that your manuscript meets PLOS ONE's style requirements, including those for file naming. The PLOS ONE style templates can be found at 
[https://journals.plos.org/plosone/s/file?id=wjVg/PLOSOne_formatting_sample_main_body.pdf](https://urldefense.proofpoint.com/v2/url?u=https-3A__journals.plos.org_plosone_s_file-3Fid-3DwjVg_PLOSOne-5Fformatting-5Fsample-5Fmain-5Fbody.pdf&d=DwQGaQ&c=sJ6xIWYx-zLMB3EPkvcnVg&r=HSWkRJnSki1_J2y9HfhzA-9UWpdx9WWzV4-iX60C3nk&m=TgAm8NDIvo8jeOvptVZdjAQdwc6OZ197goglMelhYMYnXUXIY9XCCD1pgA0hiDYn&s=vCtUuLGmuj4RC08NyhZ84QlpIN5w6HTp3UT_J_NKQsc&e=) and 
[https://journals.plos.org/plosone/s/file?id=ba62/PLOSOne_formatting_sample_title_authors_affiliations.pdf](https://urldefense.proofpoint.com/v2/url?u=https-3A__journals.plos.org_plosone_s_file-3Fid-3Dba62_PLOSOne-5Fformatting-5Fsample-5Ftitle-5Fauthors-5Faffiliations.pdf&d=DwQGaQ&c=sJ6xIWYx-zLMB3EPkvcnVg&r=HSWkRJnSki1_J2y9HfhzA-9UWpdx9WWzV4-iX60C3nk&m=TgAm8NDIvo8jeOvptVZdjAQdwc6OZ197goglMelhYMYnXUXIY9XCCD1pgA0hiDYn&s=zwNuUIyUYeul9wuNAiSMz6Vcp8YAWTnzUjLttrwNxac&e=)

We have updated the format of the tables, authors’ affiliation, and manuscript body.

2. We note that the grant information you provided in the ‘Funding Information’ and ‘Financial Disclosure’ sections do not match.

When you resubmit, please ensure that you provide the correct grant numbers for the awards you received for your study in the ‘Funding Information’ section.

We couldn’t update the Financial Disclosure section online. Our funding source is the following:

Funding: UF STEM Translational Communication Center and UF Clinical and Translational Science Institute, awarded to AM. The funders had no role in study design, data collection and analysis, decision to publish, or preparation of the manuscript.

3. Thank you for stating the following in your Competing Interests section:  
(No authors have competing interests.)

Please complete your Competing Interests **on the online submission form** to state any Competing Interests. If you have no competing interests, please state "The authors have declared that no competing interests exist.", as detailed online in our guide for authors at [http://journals.plos.org/plosone/s/submit-now](https://urldefense.proofpoint.com/v2/url?u=http-3A__journals.plos.org_plosone_s_submit-2Dnow&d=DwQGaQ&c=sJ6xIWYx-zLMB3EPkvcnVg&r=HSWkRJnSki1_J2y9HfhzA-9UWpdx9WWzV4-iX60C3nk&m=TgAm8NDIvo8jeOvptVZdjAQdwc6OZ197goglMelhYMYnXUXIY9XCCD1pgA0hiDYn&s=Tqtk8iyKMxnY0t8MxYRQXVfnBsKjuNONc2xnnVy6JIU&e=) 
 This information should be included in your cover letter; we will change the online submission form on your behalf.

The authors have declared that no competing interests exist.

4. We note that you have stated that you will provide repository information for your data at acceptance. Should your manuscript be accepted for publication, we will hold it until you provide the relevant accession numbers or DOIs necessary to access your data. If you wish to make changes to your Data Availability statement, please describe these changes in your cover letter and we will update your Data Availability statement to reflect the information you provide.

We would like to change our Data Availability Statement to the following:

Data cannot be shared publicly because of confidentiality and privacy concerns. The University of Florida Institutional Review Board approved the study, and the consent document the IRB approved assured participants that their data would not be shared beyond the research team and as aggregated in publications.

5. Please include captions for your Supporting Information files at the end of your manuscript, and update any in-text citations to match accordingly. Please see our Supporting Information guidelines for more information: [http://journals.plos.org/plosone/s/supporting-information](https://urldefense.proofpoint.com/v2/url?u=http-3A__journals.plos.org_plosone_s_supporting-2Dinformation&d=DwQGaQ&c=sJ6xIWYx-zLMB3EPkvcnVg&r=HSWkRJnSki1_J2y9HfhzA-9UWpdx9WWzV4-iX60C3nk&m=TgAm8NDIvo8jeOvptVZdjAQdwc6OZ197goglMelhYMYnXUXIY9XCCD1pgA0hiDYn&s=0jg9RqNXqo_nIsUA8JHq4x61H5RWYjhgec5M7I7xJF8&e=).

Captions for supporting information were added to the end of the manuscript and in-text citations were updated accordingly.

6. Please review your reference list to ensure that it is complete and correct. If you have cited papers that have been retracted, please include the rationale for doing so in the manuscript text, or remove these references and replace them with relevant current references. Any changes to the reference list should be mentioned in the rebuttal letter that accompanies your revised manuscript. If you need to cite a retracted article, indicate the article’s retracted status in the References list and also include a citation and full reference for the retraction notice

Reference list was updated.

Response to reviewers

**Reviewer #1: - There is a lack of consideration for the relationship between researcher/s and participants. Were all 3 researchers present at the interviews? If so, this brings a possible power balance and intimidation that may affect responses, which was not considered within the limitations. Was a relationship / rapport built with participants prior to interviewing? 3 interviewers would be an even larger issue if not, if so this should be reported.**

Thank you for the opportunity to add further clarity. Two researchers were present mostly at all times. The researcher (AE) served as the moderator for the interviews while one of the two research assistants acted as an observer and note-taker. This is now clarified on page 7, lines 147-149.

Relationship with participants:
There was no pre-interview activity or contact between the participants and interviewers. Participants were not given information about the interviewer beyond a brief introductory statement at the beginning of the interviews that described the interviewer’s role in the study and status (PhD candidate at the University of Florida).

This is now clarified on page 7, lines 153-155. The protocol was IRB approved and standard protocol for social and behavioral research that are acceptable to participants were used.

We have also uploaded a COREQ checklist for qualitative research in supporting materials (See S2 Appendix).

-It would be beneficial to see the interview schedule, and how it changed as a result of feedback from public health / nutrition professionals and a trial run with participants (what were the 'edits' you employed?).

The interview schedule was uploaded (See S1 Appendix).

We have also decided to take out the following sentence: “To improve the conversational flow and ensure clarity, applicability, and feasibility, the interview guide was tested with the population of interest” upon reflection and considering the reviewer comments regarding the edits that were made. The changes were minor overall (minimal language/grammar changes).

We have added the “population of interest” to the following sentence “The moderator followed a semi-structured interview script that was developed by the research team after a thorough review of the food security and food assistance literature and reviewed by the population of interest and qualified faculty with topic expertise in nutrition, public health, and health communication”. We feel this more accurately reflects the process without implying to the reader that the changes based on the student review were such that they would denote more expansive explanation.

**-One issue is that although the authors are noted to have attended training on the ethical conduct of research including researcher bias, these potential biases are not specified.**

Thank you for your comment. We have clarified the sentence and cited the trainings, page 7, lines 150-152.

**-It would be useful to see more consistent description of the analysis approach used for the interviews. In the abstract (which is very clear and thorough) you describe content analysis, but within the methodology this terminology is not used, and instead is described as a mixture of inductive and deductive (line 187). More clear reporting on the theoretical background (if any) of the analysis approach, and consistency across abstract and methodology, is needed.**

Thank you for pointing this out. We misnamed the approach used in the previous version of the manuscript. Edits were implemented to describe the inductive, semantic thematic analysis used to guide data analysis. This is now described on page 9, line 205. The abstract was also updated to reflect the same approach.

**-Where are the field notes? And data for the background information of the sample**?

Data on the background information of the sample can be found in the characteristics of study participants, page 11, lines 234-241 and table 1. This information was also added to the COREQ checklist.

In health-related social science, we take interview notes which are usually collected by the second researcher present (research assistant in our study). These are reported on page 7, lines 148-149.
